# Effect of Post-Sintering Shot Peening Treatment on the Corrosion Behavior of Alumix 321 Powder Metallurgy Alloy in 3.5 wt.% NaCl Solution

**DOI:** 10.3390/ma19102035

**Published:** 2026-05-13

**Authors:** Abdulwahab Ibrahim, Paul Bishop, Georges Kipouros

**Affiliations:** 1 Department of Mechanical Engineering, College of Engineering and Technology, University of Doha for Science and Technology, Al Tarafa, Jelaiah Street, Doha P.O. Box 24449, Qatar; 2 Faculty of Engineering, Department of Mechanical Engineering, Dalhousie University, 5248 Morris Street, Halifax, NS B3H 4R2, Canada; paul.bishop@dal.ca; 3 Department of Chemical and Biological Engineering, College of Engineering, University of Saskatchewan, 57 Campus Dr, Saskatoon, SK S7N 5A9, Canada

**Keywords:** Alumix 321, powder, corrosion, shot peening, cyclic polarization, stair step polarization

## Abstract

The growing emphasis on environmental sustainability and the need for advanced manufacturing methods have accelerated progress in material processing. Aluminum powder metallurgy (APM) is particularly promising due to aluminum’s low density, high strength-to-weight ratio, and the inherent benefits of the powder metallurgy (PM) process. However, the corrosion resistance of sintered aluminum components remains a significant concern. In this study, shot peening (SP) was employed as a surface modification technique to improve the corrosion behavior of Alumix 321 PM alloy. Samples of the as-sintered and shot-peened Alumix 321 PM alloy, together with the wrought alloy counterpart AA6061, were characterized using non-contact optical profilometry, optical microscopy (OM), and scanning electron microscopy (SEM). Corrosion performance was evaluated in 3.5 wt.% NaCl solution using Tafel extrapolation (TE), cyclic polarization (CP), stair step polarization (SSP), and electrochemical impedance spectroscopy (EIS). The results revealed that shot peening increased surface roughness and significantly reduced the corrosion rate from 0.079 mmpy to 0.004 mmpy for the unpeened and peened samples, respectively. While pitting was the dominant corrosion mechanism in the wrought alloy, the PM alloy exhibited a combination of pitting, crevice, and intergranular corrosion. These findings highlight the potential of SP in enhancing the durability of aluminum-based PM components, offering valuable insights for industrial applications.

## 1. Introduction

The growing emphasis on environmental sustainability continues to drive innovations in alloy production and advanced manufacturing methods. Among these, powder metallurgy (PM) has gained increasing attention as a promising technique for producing aluminum components with enhanced properties and reduced environmental impact. This development in manufacturing techniques is critical in meeting the stringent demands of high-strength applications, as evidenced by recent studies on the processing of aluminum powder metallurgy (APM) alloys [1,2,3,4,5,6,7].

APM, in particular, has emerged as an effective approach due to the processing advantages of PM combined with the intrinsic benefits of aluminum. However, a major limitation to the broader application of APM components is their inferior corrosion resistance, primarily attributed to residual porosity remaining after the sintering process. This porosity significantly compromises the integrity and durability of aluminum PM parts in corrosive environments, making corrosion behavior a critical factor in the design and performance of such components [8,9,10,11,12,13].

Judge and Kipouros [14] reported that APM alloys generally exhibit lower corrosion resistance than their wrought and cast counterparts. They attributed the inferior performance to the high surface area and interconnected open porosity inherent in sintered structures. To achieve corrosion resistance comparable to wrought alloys, further densification and post-processing treatments are necessary. Density and porosity are key parameters influencing both mechanical and corrosion behavior in APM alloys. Accurate measurement of these features is essential for optimizing performance. Steedman et al. [15] evaluated two techniques, helium pycnometry and oil impregnation, for determining the density of aluminum–silicon PM alloys (Alumix 231 and Dal 6-Si). Their study concluded that helium pycnometry provides more precise results, as conventional oil impregnation fails to account for fine or inaccessible pores. Their work also emphasized the need for improved characterization techniques to quantify pore volume, surface area, and porosity distribution. These factors are critical for understanding corrosion behavior in porous PM alloys. Surface modification and post-sintering treatments are widely used to mitigate the effects of porosity and enhance corrosion resistance in APM materials. Techniques such as anodizing, surface coating, resin impregnation, and heat treatment can alter microstructure, seal pores, and redistribute secondary phases, thus improving corrosion resistance and mechanical performance. The choice of treatment depends on the targeted application and desired property profile [14,16,17].

Among surface treatments, SP is a well-established mechanical surface modification technique, commonly employed to improve the fatigue resistance of wrought aluminum alloys [18,19,20]. Prior work on aluminum PM alloys has applied SP across a range of intensities and media sizes and has reported improved surface integrity and fatigue performance, often attributed to near-surface densification and compressive residual stress. For instance, Zupanc and Grum [21] demonstrated that SP treatment on 7075-T651 aluminum alloy increased fatigue threshold stress and reduced surface pitting. The application of SP has also been utilized in the domain of PM materials. Studies have shown that SP can improve fatigue strength by as much as 40% in PM carbon steels with 1.5 wt.% carbon content [22].

Despite its proven benefits, limited research exists on the influence of shot peening on the corrosion and mechanical behavior of APM alloys. Notably, Lynen et al. [23] reported a 10–15% improvement in fatigue strength in Alumix 431 APM alloy following SP at intensities of 12 A and 16 A. Harding et al. [8] investigated the effect of SP on an Al-Zn-Mg-Cu PM alloy, revealing the development of significant compressive residual stresses (−293 MPa), which contributed to a 29% increase in fatigue strength. These findings highlight the potential of shot peening as a post-processing technique for enhancing the performance of APM components. However, its effect on the corrosion behavior of aluminum-based PM alloys, particularly Alumix 321, remains underexplored as most of the prior studies primarily concentrate on the corrosion resistance of wrought aluminum alloys [24,25,26,27]. The corrosion response of porous press-and-sinter aluminum powder metallurgy (APM) cannot be assumed to follow trends reported for wrought AA6061 because corrosion in APM is strongly governed by pore connectivity (crevice-like attaching), and micro galvanic coupling with second-phase particles. Accordingly, the novelty of this work is threefold: (i) it focuses on the post-sintering shot peening of Alumix 321 (AA6061 equivalent chemistry) where corrosion is dominated by accessible and interconnected porosity; (ii) it combines Tafel extrapolation, cyclic polarization, stair step polarization (SSP), and EIS to separate uniform corrosion kinetics from localized corrosion susceptibility; and (iii) it links electrochemical metrics to quantified pore features and corrosion morphology in 3.5 wt.% NaCl. This study, therefore, clarifies how shot peening alters the balance between surface densification/pore sealing and roughness/heterogeneity-driven localized attack in aluminum PM alloys.

This study aims to address this gap by investigating the impact of shot peening on the corrosion characteristics of Alumix 321 PM alloy in a 3.5 wt.% NaCl solution. Accordingly, the objective of this work is to explore the mechanisms by which post-sintering shot peening alters the corrosion response of Alumix 321 PM alloy in 3.5 wt.% NaCl, specifically regarding the roles of near-surface pore sealing and surface deformation in controlling electrolyte access to interconnected porosity, passive film stability, and localized corrosion kinetics using TE, CP, SSP, and EIS.

The results demonstrate that post-sintering shot peening significantly reduces the corrosion rate of Alumix 321 PM alloy in 3.5 wt.% NaCl solution, primarily through near-surface pore deformation and partial sealing that limit electrolyte access to interconnected porosity. Electrochemical testing revealed a marked decrease in corrosion current density and a delayed localized corrosion initiation under stair step polarization conditions. While conventional cyclic polarization indicated only modest changes in pitting potential, the combined use of TE, SSP, and EIS confirmed that shot peening improves overall corrosion kinetics in the porous PM alloy. These findings clarify the role of surface densification and pore accessibility in controlling corrosion behavior of aluminum powder metallurgy systems.

## 2. Materials and Methods

### 2.1. Materials

**Figure 1:**
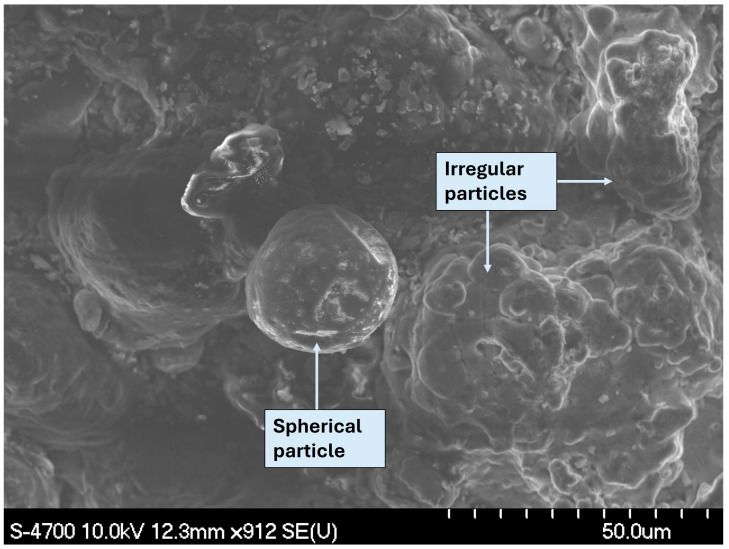
SEM micrograph shows the morphology of the as-received Alumix 321 powder.

**Figure 2:**
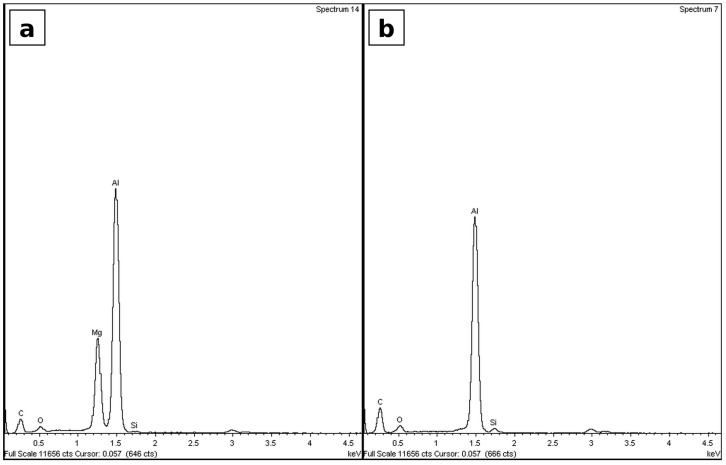
EDS spectra acquired from: (**a**) spherical Alumix 321 particles; (**b**) irregular Alumix 321 particles.

All experiments were conducted using a commercially available powder metallurgy alloy known as Alumix 321. This alloy is chemically equivalent to the AA6061 wrought alloy and was supplied by Ecka Granules as a pre-mixed formulation containing 1.5% Licowax C as a lubricant. Chemical composition of the Alumix 321 powder was determined using Atomic Absorption Spectroscopy (AA). The chemical analysis results for the Alumix 321 PM alloy and its AA 6061 counterpart are presented in Table 1. Further morphological and compositional analyses were carried out using a Hitachi S-4700 Cold Field Emission Scanning Electron Microscope (SEM). The powder particles exhibited shapes ranging from spherical to irregular. This morphological variation is attributed to differences in the powder production techniques. Particle size distribution (PSD) of the Alumix 321 raw powder was determined from SEM micrographs using image analysis. The resulting PSD percentiles were D10 = 12.16 µm, D50 = 21.02 µm, and D90 = 41.11 µm. Energy Dispersive X-ray Spectroscopy (EDS) indicates that some particles are Al-rich (with Si-bearing constituents), whereas other particles are Mg-enriched. Because EDS is compositional and not crystallographic, these features are described as Mg-enriched regions rather than ‘pure Mg’ particles. SEM micrographs and corresponding EDS results are shown in Figure 1 and Figure 2, respectively.

### 2.2. Powder Metallurgy Processing

All powder metallurgy (PM) samples were fabricated using the conventional press-and-sinter method, which involves compacting a powder mixture in a die followed by sintering under a controlled atmosphere using liquid phase sintering (LPS). The pre-lubricated Alumix 321 powder was first blended in a Turbula T2-F mixer (WAB-GROUP, Muttenz, Switzerland) for 20 min to ensure uniform homogeneity. The mixture was then compacted uniaxially using an Instron SATEC 5594-200 HVL (Instron Industrial Products Group, Grove city, PA, USA) compression frame at a pressure of 500 MPa to form cylindrical pucks (15 mm diameter) from 2.5 g of powder. These pucks were subsequently used for density measurements, shot peening, and corrosion testing.

**Figure 3:**
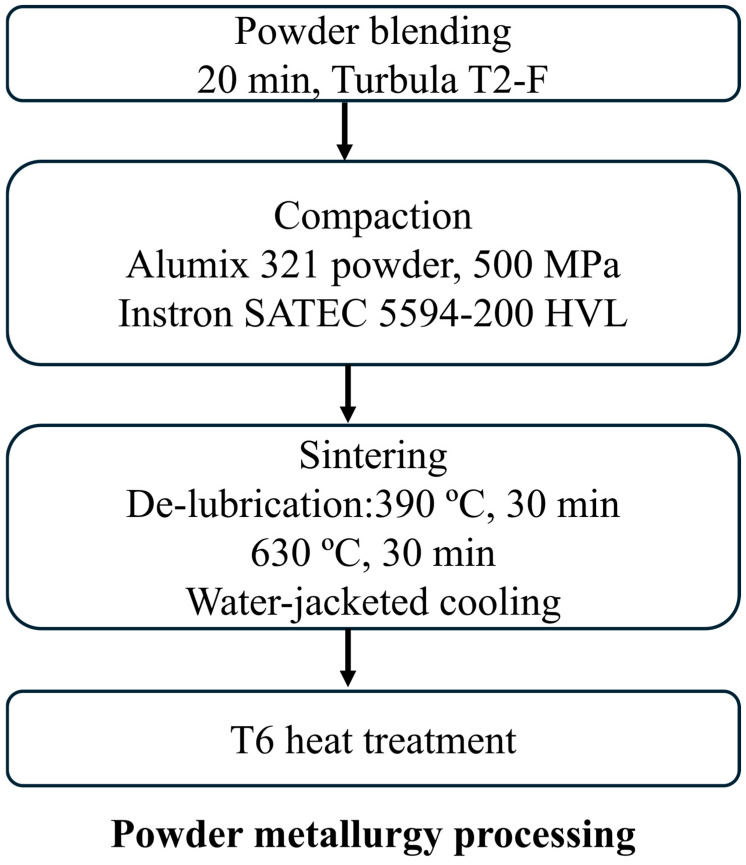
Schematic diagram of powder metallurgy processing.

Sintering was conducted in a bell jar furnace (Materials Research Furnaces, MRF) and consisted of three stages: de-lubrication, sintering, and cooling. The process was performed under a continuous flow of ultra-high-purity nitrogen gas (>99.999%) to prevent oxidation. The samples were first held isothermally at 390 °C for 30 min to remove the lubricant, followed by sintering at 630 °C for 30 min. Post-sintering cooling was carried out in a water-jacketed section of the furnace to control the cooling rate. Figure 3 shows a schematic diagram of the powder processing. All sintered Alumix 321 PM samples underwent a T6 heat treatment to enhance their mechanical properties. A detailed sintering protocol for this alloy system is available in our previous publication on the sinterability of the Alumix 321 PM alloy [28].

### 2.3. Shot Peening Treatment

Following sintering, the 15 mm pucks underwent secondary processing consisting of heat treatment and shot peening. The heat treatment followed a T6 tempering schedule, beginning with solution treatment at 530 °C for 1 h, water quenching, and artificial aging at 160 °C for 18 h. After heat treatment, the sintered samples were subjected to shot peening using a Cana Blast machine manufactured by International Surface Technology (IST Inc., Hsinchu City, Taiwan). Prior to peening, the samples were ground, polished, and then cleaned in an ultrasonic bath. The shot peening system consists of a closed peening cabinet designed to precisely control the shot flow. The sample holder was mounted on a movable aluminum ram driven by an electric motor and controlled via an automated actuator system to ensure consistent coverage. Prior to performing the shot peening on the PM samples, standard Almen strips were used to calibrate the system. The arc height of the strips was measured to determine the Almen intensity of the process. Zirconium oxide beads with a diameter of 300 μm were employed as the peening medium. The operating parameters were selected to achieve a peening intensity of 0.4 mmN. The peening media and size, nozzle–specimen distance, impingement angle, exposure time, and specimen motion configuration were maintained constant for all treated samples to ensure reproducible surface deformation. The achieved coverage was approximately 95% and was verified by surface image analysis. These parameters represent the primary practical descriptors required for replication of the SP treatment in the present work.

### 2.4. Surface and Metallographic Characterization

The surface characteristics of the as-sintered, shot-peened, and AA 6061-T6 samples were evaluated using a non-contact Nanovea Micro-Profiler (Model PS50) equipped with a 1.2 mm sensor and a resolution of 0.1 µm. Full surface scans of the disk specimens were performed, and the data were analyzed using the Nanovea 3D software package version 6.

Microstructural examinations were conducted on sintered, shot-peened, and AA 6061 samples, as well as on corroded specimens. Mounted samples were ground and polished using a series of diamond pastes ranging from 9 µm to 0.1 µm, followed by rinsing with distilled water and drying. An Olympus BX51-TRF optical microscope (Nagano Olympus Co., Ltd., Ina, Japan) integrated with an Olympus DP71 digital camera (Nagano Olympus Co., Ltd., Ina, Japan) was employed for optical imaging.

High-resolution surface and microstructural analyses were further performed using a Hitachi S4700 Cold Field Emission Scanning Electron Microscope (FESEM) (Hitachi High Tech Corporation, Hitachinaka, Japan). This included examination of both corroded and uncorroded surfaces of the sintered, shot-peened, and AA 6061-T6 samples. Elemental composition and qualitative phase identification were carried out using Energy Dispersive X-ray Spectroscopy (EDS) with an Oxford X-Sight 7200 system (Oxford Instruments NanoAnalysis, Buckinghamshire, UK). EDS analysis was particularly utilized to investigate the phases present in the sintered samples and to assess the composition of corrosion products on the degraded surfaces.

### 2.5. Corrosion Experiments

Corrosion behavior was evaluated using an EG&G PARC Model 273A potentiostat (Princeton Applied Research, Oak Ridge, TN, USA) controlled via CorrWare software version 3.2. Prior to use, the system was calibrated using a laboratory-fabricated “dummy” cell. Electrochemical tests were performed in a standard three-electrode bulb cell configuration. The working electrode (sample) potential was measured against a saturated calomel electrode (SCE) via a Luggin capillary, while a large graphite rod (area = 3.85 cm^2^) served as the counter electrode.

All experiments were conducted in a synthetic electrolyte composed of 3.5 wt.% NaCl (analytical grade) dissolved in deionized water. The solution was naturally aerated, maintained at a constant temperature of 22 ± 1 °C, and its pH was kept between 6.2 and 6.6. A custom Teflon sample holder was designed to accommodate specimens of 1.5 cm diameter, exposing 1.0 cm^2^ of the surface to the test solution.

The materials tested included wrought AA 6061 alloy, sintered Alumix 321 PM alloy, and shot-peened Alumix 321 PM alloy. Wrought samples were sectioned from a 15 mm diameter extruded rod perpendicular to the extrusion direction. Sintered and shot-peened samples were used in their original disk form (15 mm diameter), with no additional machining required. Prior to testing, the wrought and sintered samples were prepared using conventional metallographic procedures. In contrast, shot-peened samples were tested as-is to preserve surface conditions resulting from peening.

Before electrochemical measurements, samples were immersed in the electrolyte to allow for the stabilization of the open circuit potential (OCP): 2 h for sintered samples and 1 h for wrought ones. Potentiodynamic polarization tests were employed to investigate electrochemical behavior. Tafel extrapolation (TE), cyclic polarization (CP), and stair step polarization (SSP) techniques were used to evaluate corrosion current density, pitting susceptibility, and pit propagation under stepped conditions, respectively. A scan rate of 0.1667 mV/s was used for both TE and CP tests. For SSP tests, a step size of 10 mV and a step duration of 2500 s were applied.

Electrochemical impedance spectroscopy (EIS) was conducted using a Gamry 600 poteniostat (Gamry Instruments, Inc., Warminster, PA, USA) with a flat cell configuration. Experiments were conducted using Gamry’s Electrochemist software version 5.0, and data fitting and circuit modeling were performed using Chemo Plot version 5.0. EIS measurements were performed over a frequency range of 100 kHz to 10 mHz, with a 10 mV AC amplitude superimposed on the OCP. Prior to each EIS test, samples were allowed to stabilize in the electrolyte for 1 h to ensure a steady-state OCP was established.

## 3. Results and Discussion

### 3.1. Surface Topography Assessment

**Figure 4:**
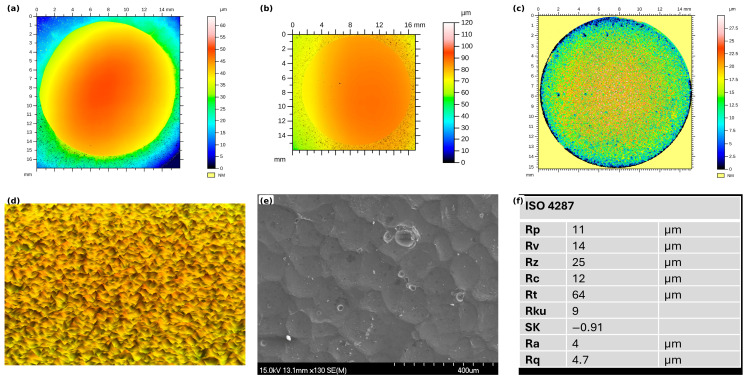
Schematic illustration of the test samples’ surface analysis: (**a**) AA 6061 alloy; (**b**) unpeened Alumix 321 PM alloy; (**c**) shot-peened Alumix 321 PM alloy; (**d**) 3D surface topography; (**e**) real surface, and (**f**) surface roughness parameters.

Shot peening (SP) significantly altered the surface topography of the tested samples. Figure 4 presents a comparative surface analysis of the three conditions. Figure 4a shows the surface of the wrought AA 6061-T6 alloy, while Figure 4b displays the as-sintered Alumix 321 PM alloy, where residual surface porosity is clearly visible. Figure 4c illustrates the shot-peened Alumix 321 PM alloy, characterized by a distinct dimpled morphology resulting from plastic deformation induced by the peening media. The corresponding three-dimensional surface topography is shown in Figure 4d, where color coding represents height variation across the scanned area (blue indicating lower elevations and red indicating higher features). Figure 4e presents the real surface image, and Figure 4f summarizes the extracted surface roughness parameters. These figures collectively demonstrate the pronounced morphological modification induced by shot peening on the PM alloy surface.

**Figure 5:**
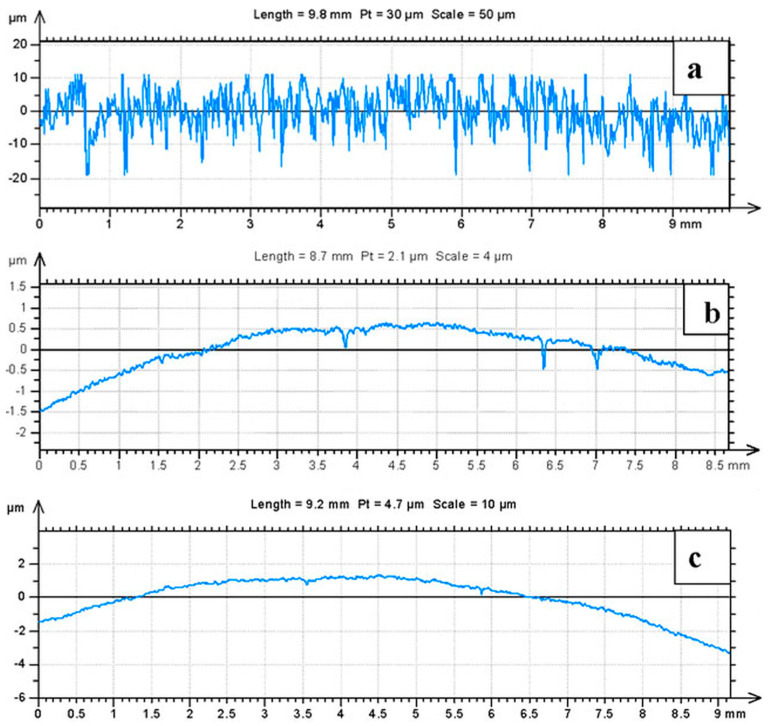
Profile scan of: (**a**) Alumix 321—peened; (**b**) Alumix 321 PM—unpeened; (**c**) wrought AA 6061 alloy [30].

Figure 5 displays the surface profile scans of three sample types: wrought AA 6061-T6, as-sintered Alumix 321 PM, and Alumix 321 PM peened to an intensity of 0.4 mmN. Surface roughness measurements were extracted from the profile data. The results showed consistent agreement with the visual output of the micro profilometer.

For both the as-sintered and wrought samples, the surface roughness values were nearly identical at 0.033 ± 0.003 µm and 0.034 ± 0.004 µm, respectively. This similarity can be attributed to the absence of any surface modification and the use of a common metallographic preparation procedure. In contrast, the roughness of the shot-peened samples was significantly higher, reaching 3.95 ± 0.252 µm. This marked increase confirms the substantial surface deformation introduced by the peening process. It should be noted that the color scales in the 3D surface maps represent the full height variation over the scanned area (including waviness), whereas the reported roughness values (Ra) were calculated after surface leveling and ISO 4287 filtering; therefore, the Z range in the maps should not be interpreted as Ra [29].

### 3.2. Microstructural Evaluation

**Figure 6:**
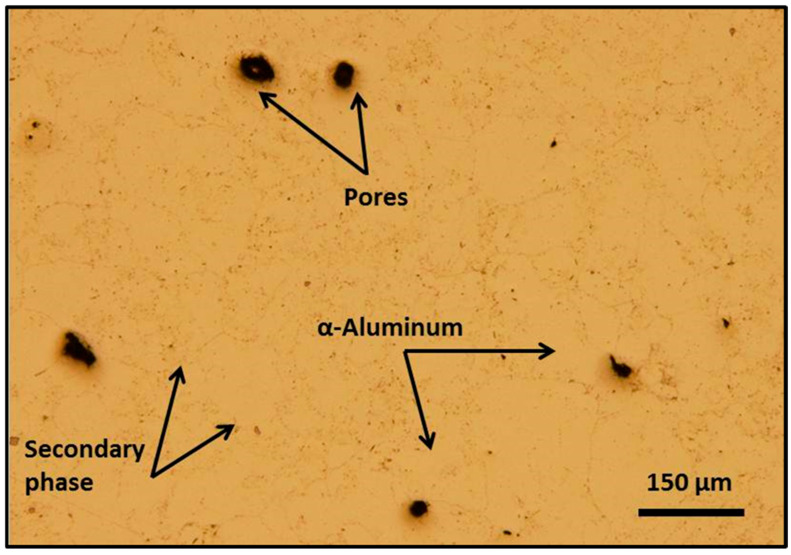
Optical micrograph of Alumix 321 in as-sintered material condition.

**Figure 7:**
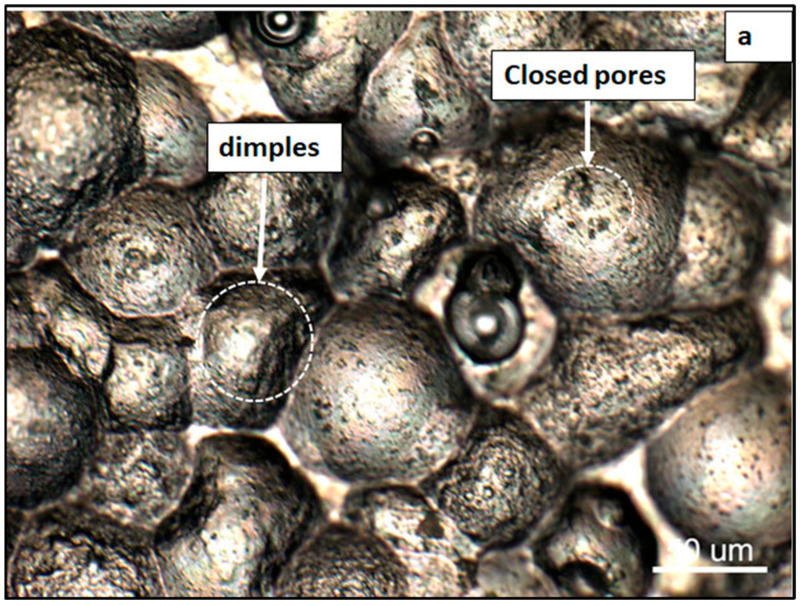

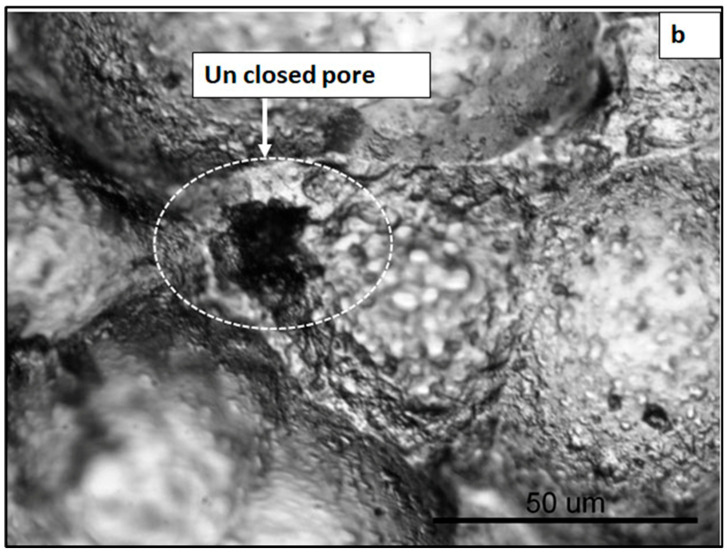
High-magnification optical micrographs of shot-peened Alumix 321 PM alloy: (**a**) dimple microstructure; (**b**) retained porosity after peening.

The densification achieved after compaction and sintering is notably high, as illustrated in Figure 6. The microstructure is dominated by the aluminum α-phase, with visible precipitates along the grain boundaries. These features are characteristic of sintered materials, including the presence of closed and rounded pores, signifying effective densification. This enhanced densification can be attributed to the combined effect of the high compaction pressure (500 MPa) and liquid-phase sintering (LPS), which promotes efficient mass transport within the alloy matrix [31,32]. As a result, the density increased from 2.576 g/cm^3^ in the green compact to 2.645 g/cm^3^ after sintering, corresponding to a relative density of 98.4% of the theoretical density (TD). The effect of shot peening on porosity was assessed qualitatively by optical and SEM observations, which show deformation and partial closure of surface-connected pores. Figure 7a presents a high-magnification optical micrograph revealing the effects of shot peening on the microstructure of the Alumix 321 PM alloy. The image displays a dimple-like surface morphology, generated by the intense mechanical impact of the shot peening process. Although shot peening can deform and partially close surface voids, residual porosity remains evident. These pores are often irregular, interconnected, and extend beneath the modified surface layer. Figure 7b further shows that smaller pores undergo significant deformation and partial closure, which reduces their size and mitigates their role as potential stress concentrators and corrosion initiation sites.

**Figure 8:**
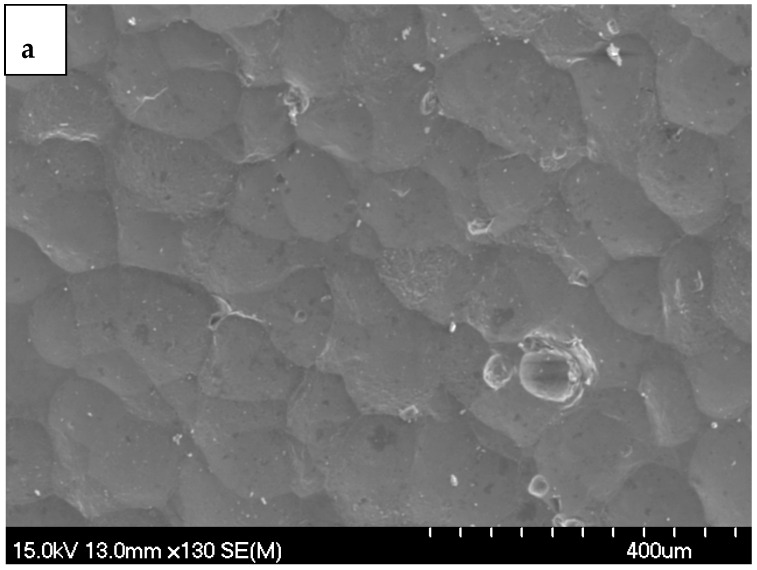

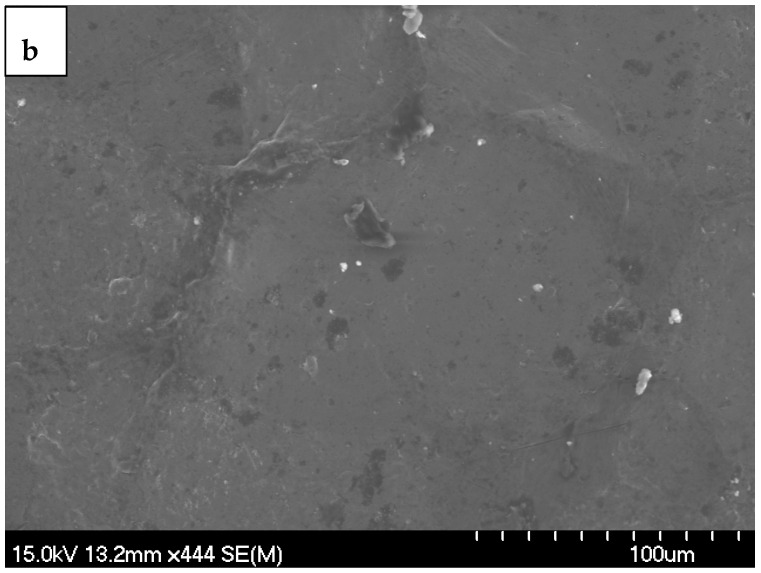
SEM micrograph of Alumix 321 PM alloy: (**a**) low magnification shows dimple structure; (**b**) high magnification shows sealing of possible pores.

Figure 8a,b present SEM micrographs of the shot-peened Alumix 321 PM alloy, clearly demonstrating the alteration in surface topography due to the peening process. The observed dimple structure appears as valleys and peaks and is a direct result of the impact from the peening media. While the literature reports that shot peening may introduce microcracks that can compromise material performance [33,34], no surface microcracks were observed at the magnifications examined. However, subsurface microcracks cannot be ruled out without high-magnification cross-sectional SEM and/or microhardness profiling. Accordingly, we limit our claim to the observed surface condition.

### 3.3. Electrochemical Behavior

**Figure 9:**
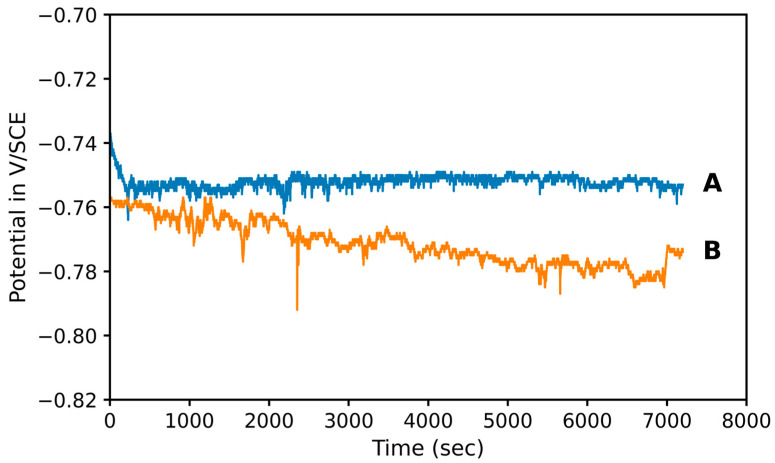
OCP variation as a function of time of Alumix 321 PM alloy before (A) and after shot peening (B).

OCP measurements were performed on Alumix 321 PM samples before and after the application of shot peening. The variation of potential versus time is presented in Figure 9. The time dependence of OCP reflects stabilization of the surface oxide/hydroxide film and progressive conditioning of the electrode/electrolyte interface (including wetting of surface-connected porosity). Shot peening modifies surface morphology and pore accessibility, leading to a different OCP evolution pathway before reaching a quasi-steady potential. The shot-peened samples exhibited a more active electrochemical potential, as evidenced by a lower E_ocp_ compared to the as-sintered condition. Specifically, the measured OCP values were −0.753 ± 0.005 V/SCE for the as-sintered samples and −0.769 ± 0.020 V/SCE for the shot-peened counterparts. This more negative potential of the peened samples compared to the unpeened is attributed to the increased surface energy and residual stress introduced by mechanical deformation during shot peening, which alters surface characteristics and promotes higher reactivity [35,36].

**Figure 10:**
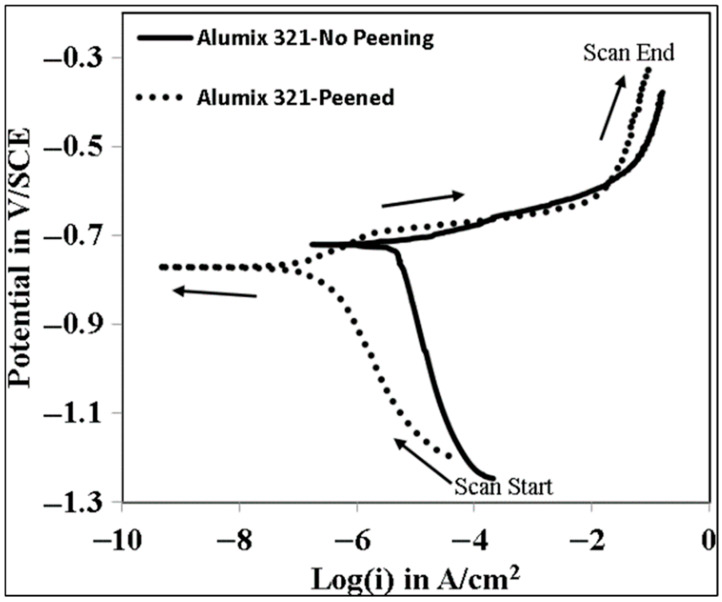
Tafel extrapolation plot of Alumix 321 PM alloy before and after shot peening.

To assess the corrosion kinetics, TE tests were conducted. Figure 10 illustrates the polarization curves for both peened and unpeened specimens. The polarization response can be divided into two distinct regimes: a near-corrosion-potential region and a higher anodic potential region ranging from approximately −0.6 to −0.4 V/SCE. Notably, the unpeened samples exhibited a sharp current rise near their corrosion potential (−0.753 V/SCE), indicating early pitting initiation. This is verified by the calculated ΔE (ΔE = Ecorr − Epit), which was ~0.00 V, implying a negligible passivation range and confirming pitting initiation at Ecorr. In contrast, the shot-peened samples exhibited a delayed pitting onset at −0.716 V/SCE, resulting in a ΔE of 0.046 V. A large difference between the two values usually indicates a wider passive region and possibly a better pitting resistance, signifying improved resistance to localized corrosion [37].

The second polarization zone, corresponding to potentials between −0.6 and −0.4 V/SCE, is characterized by current densities in the order of 10^−2^ A/cm^2^. In this region, the anodic curve of both peened and unpeened samples converge, indicating the breakdown of the passive film and the onset of active metal dissolution. At these higher anodic potentials, the similarity in current response suggests that passivity is lost for both conditions, and the corrosion behavior becomes dominated by uniform metal dissolution rather than localized attack. The separation between the samples near Ecorr is dominated by differences in the cathodic response, indicating that surface condition (oxide/hydroxide characteristics and accessible reaction area) strongly influences oxygen reduction kinetics. Shot peening promotes a more heterogeneous near-surface film and reduces electrolyte access to surface-connected porosity.

**Figure 11:**
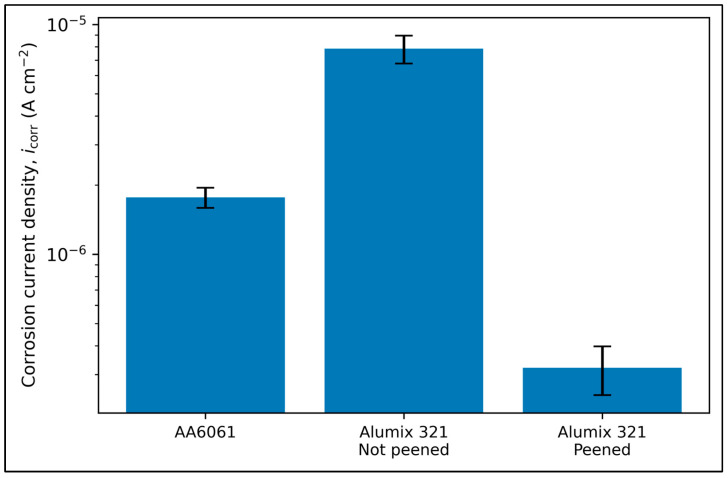
Comparison of the corrosion current of sintered unpeened Alumix 321, peened Alumix 321, and wrought AA 6061.

Shot peening also markedly influenced the corrosion current density, reducing it from 7.86 × 10^−6^ A/cm^2^ (unpeened) to 3.20 × 10^−7^ A/cm^2^ (shot-peened), which corresponded to a corrosion rate reduction from 0.079 mmpy to 0.004 mmpy. These values indicate a substantial improvement in corrosion resistance. Interestingly, the corrosion current of the shot-peened samples was even lower than that of wrought AA 6061, as shown in Figure 11. This enhancement is attributed to surface densification, reduction in accessible porosity, and potential enhancement of passive film formation. Furthermore, the possible effect on porosity and probable closing off of the interconnected pores caused by peening may have contributed to better corrosion resistance by minimizing the likelihood for localized corrosion sites. It is worth mentioning that residual stresses were not measured in this work; the dominant corrosion improvement in the porous PM alloy is consistent with reduced electrolyte access to interconnected porosity due to near-surface deformation and pore closure. Compressive stress effects may contribute but are not claimed as the sole mechanism.

One of the main challenges in PM alloys is the presence of open and interconnected porosity, which adversely affects both mechanical and corrosion performance. These pores act as micro crevices, promoting differential aeration cells that are highly conducive to localized corrosion [13]. When exposed to a chloride-containing environment (e.g., 3.5 wt.% NaCl), these crevices can trap stagnant electrolytes, creating acidic micro environments rich in chloride ions that prevent stable passivation. Porosity in PM materials is categorized into isolated and interconnected pores [38]. The interconnected pores are particularly problematic for corrosion resistance since these pores form networks and offer an extensive pathway for corrosion reactions to accelerate. Shot peening mechanically deforms the surface, effectively sealing many of the interconnected pores and thus impeding the access of the electrolyte into the bulk. This sealing effect likely plays a fundamental role in the observed enhancement in corrosion resistance.

**Figure 12:**
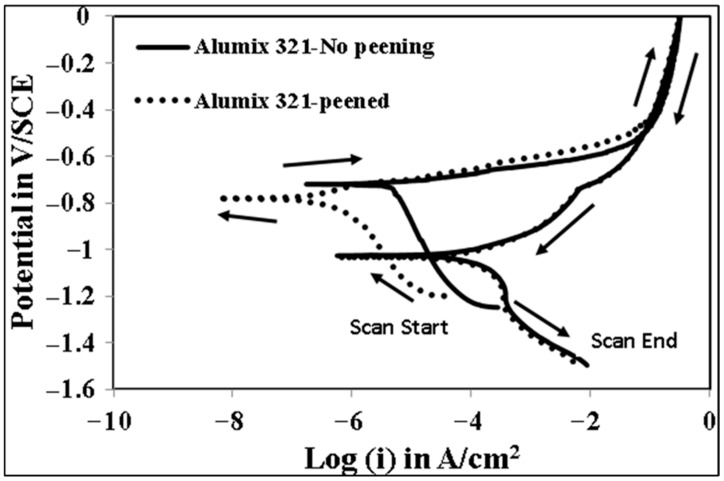
Cyclic polarization plot of peened and unpeened Alumix 321 PM alloy in 3.5 wt.% NaCl solution.

Aluminum and its alloys, particularly Al-Mg-Si, are known for their susceptibility to localized corrosion [39,40,41,42,43]. To evaluate the tendency for pitting and crevice corrosion in Alumix 321 PM alloy, both peened and unpeened samples were tested. Figure 12 shows the cyclic polarization curves for both shot-peened and unpeened samples. The shot-peened samples exhibited a more gradual increase in anodic current density, indicating improved passivity. Although both conditions showed a sharp rise in current density, indicating pitting initiation, this transition occurred at a slightly higher potential in the peened samples (−0.716 ± 0.011 V/SCE) compared to the unpeened samples (−0.714 V/SCE). At high anodic polarization, both surfaces are driven beyond the stability of the passive film and the curves converge, indicating similar active dissolution once breakdown is enforced. The relevant corrosion rate improvement is, therefore, drawn primarily from the near-Ecorr region (icorr) rather than from the highly anodic regime. The re-passivation potential remained nearly identical for both conditions at −1.050 ± 0.001 V/SCE. The corrosion potential (E_corr_) of the shot-peened samples was also more negative (−0.762 ± 0.025 V/SCE) compared to the unpeened samples (−0.719 ± 0.016 V/SCE), consistent with the OCP results.

In contrast to our results, Chen et al. [34] reported that shot peening shifted the E_corr_ of AA 6061 toward more positive values following shot peening, which they attributed to the formation of an ultra-fine nanocrystalline surface layer. The discrepancy is likely due to the distinct microstructural characteristics between wrought AA 6061 and the porous structure of Alumix 321 PM alloy. In the latter, the predominant effects arise from porosity modification and residual stress redistribution rather than grain refinement. This conclusion is in agreement with findings reported by other researchers [26,44]. It should be noted that the cyclic polarization (CP) results indicate only a modest change in localized corrosion susceptibility after shot peening, as the differences in the characteristic potentials (e.g., $E_{pit}$ and $E_{rep}$) are small and may fall within the experimental scatter expected for porous powder metallurgy surfaces.

**Figure 13:**
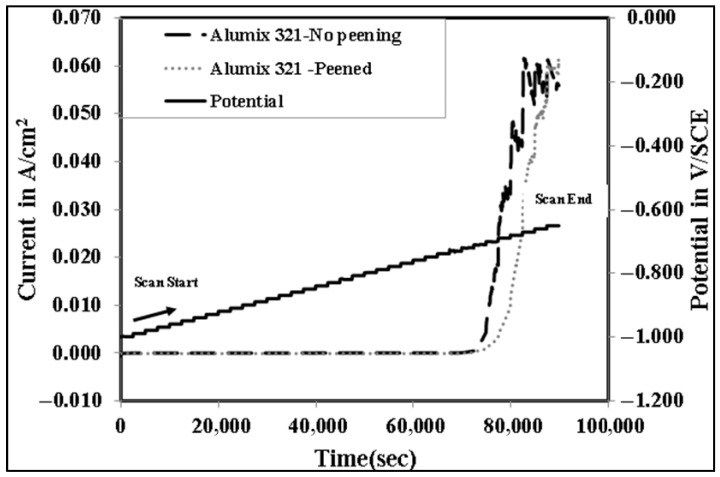
Stair step polarization plot of peened and unpeened Alumix 321 PM alloy in 3.5 wt.% NaCl solution.

The development of localized corrosion, particularly crevice corrosion, exhibits pronounced time-dependent behavior [45,46]. This aspect is critical for understanding both the extent and severity of corrosion within the micro crevices associated with the inherent porosity of APM alloys. Consequently, the long-term exposure of sintered components, such as Alumix 321 PM, must be carefully considered when evaluating their corrosion resistance and service life. Although a relatively slow scan rate of 0.1667 mV/s was employed in this study, there remains a concern that such a rate may not sufficiently capture the time-dependent nature of crevice corrosion initiation, especially within subsurface pores. To overcome this limitation, an advanced electrochemical technique utilizing an even slower scan rate was adopted to better simulate the conditions conducive to the onset and propagation of localized corrosion within porous structures. This approach enhances the reliability and accuracy of corrosion assessment, particularly in determining the susceptibility of Alumix 321 PM alloy to localized attack under prolonged exposure. For this purpose, stair step polarization (SSP) was employed. This technique is characterized by exceptionally slow potential increments with periods of approximately 41 min per step. Figure 13 presents the SSP results, illustrating both the potential variation over time and the corresponding current response for peened and unpeened Alumix 321 PM samples. At lower potentials, both sample types exhibited similarly low current densities, indicating stable passive behavior. However, at a distinct potential threshold, a sharp increase in current density was observed, signifying the initiation of localized corrosion. The threshold potential in SSP was defined operationally as the first potential step at which the current density increased sharply and remained elevated during the dwell period, indicating stable initiation/propagation of localized corrosion under near-steady conditions. Importantly, this transition occurred at a more anodic potential in the shot-peened samples (−0.690 V/SCE) compared to the unpeened ones (−0.711 V/SCE), indicating an enhancement in pitting resistance due to the surface modification. The observed difference in pitting potential underscores the sensitivity and effectiveness of the SSP technique in evaluating localized corrosion resistance, offering potentially greater resolution than conventional cyclic polarization methods. The clear separation in pitting behavior supports the argument that the extended dwell times inherent in SSP are critical for establishing the electrochemical conditions necessary for pit initiation. To the best of our knowledge, SSP has rarely been applied to porous aluminum PM alloys for localized corrosion assessment on APM alloys. Because Tafel extrapolation provides an instantaneous kinetic estimate near Ecorr and can be less representative under strongly localized attack, the corrosion rate comparison is interpreted together with CP/SSP behavior, EIS parameters, and post-corrosion surface/cross-sectional observations. Long-term immersion mass loss testing would be valuable for future work to quantify multi-day corrosion under fully natural exposure conditions. Comprehensive electrochemical parameters derived from these analyses are summarized in Table 2, offering a detailed comparison of the corrosion behavior of the investigated alloy in both peened and unpeened states.

The combined polarization results indicate that shot peening produces a clear improvement in overall corrosion kinetics (lower Icorr) while producing a more modest change in localized corrosion indicators in conventional cyclic polarization (CP). This apparent mismatch is expected for porous APM alloys. Icorr is strongly influenced by the effective electrochemically active area and electrolyte access to pore networks (micro crevice behavior), which can be reduced by pore deformation and partial sealing. In contrast, localized corrosion initiation in chloride solution is governed by the most susceptible micro sites including Fe-rich intermetallic particles acting as local cathodes, residual interconnected pores that remain accessible, and heterogeneity introduced by peening. Therefore, CP may show only a small shift in E_pit_ and little change in E_rep_ even when the uniform corrosion rate decreases substantially.

**Figure 14:**
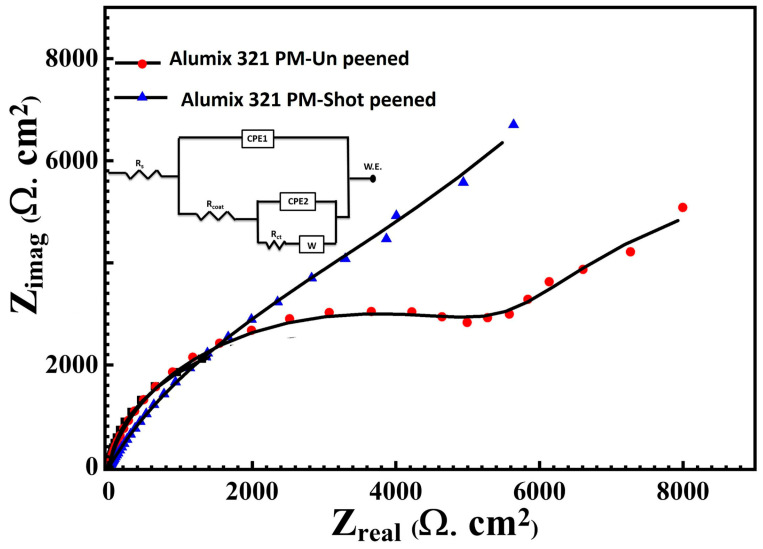
Nyquis plot for unpeened Alumix 321 PM and shot-peened Alumix 321 PM alloys.

To gain a deeper understanding of the corrosion behavior of Alumix 321 PM alloy, electrochemical impedance spectroscopy (EIS) measurements were carried out. Figure 14 presents the Nyquist plots, and the equivalent circuit parameters are summarized in Table 3. The model represents a two-time-constant equivalent circuit with a parallel configuration, commonly used to characterize the non-uniform corrosion behavior of aluminum electrodes in an electrolyte. This equivalent circuit (EC) comprises the uncompensated solution resistance (Rs), oxide film resistance (Rox), charge transfer resistance (Rct), and two constant phase elements (CPE1 and CPE2) that replace the ideal capacitive components to achieve a more accurate data fit. The non-ideal capacitive response observed in the double layer capacitance can be attributed to several factors, including surface roughness and non-uniformity of the aluminum surface or uneven current distribution. The diffusion Warburg impedance was identified by (W). The impedance behavior of the constant phase element (CPE) is mathematically represented by equation below.

$$ZQ=[Y_{o}^{-1}X{\left(jw\right)}^{-n}]$$

In this relation, *ZQ* denotes the CPE impedance (Ω·cm^−2^), and *Q* is the CPE coefficient reflecting the non-ideal capacitive characteristics of the interface. The imaginary unit is defined as $j=\sqrt{-1}$, and the angular frequency is given by *ω* = 2*πfmax*, where fmax corresponds to the frequency at which the imaginary impedance component attains its maximum value. The exponent *n* (0 ≤ *n* ≤ 1) represents the deviation from ideal capacitive behavior, which is typically associated with the degree of surface heterogeneity or roughness. Specifically, when n = 0, the CPE acts as a pure resistor, whereas n = 1 corresponds to an ideal capacitor, indicating a perfectly homogeneous surface. The Nyquist plot shows that the post-sintering shot peening process has influenced the electrochemical behavior of Alumix 321 PM alloy. The data show that the Ypo increases from 24.42 Μs·s^n^·cm^−2^ for the unpeened samples to 155.8 μS·s^n^·cm^−2^ for the peened sample, reflecting the impact of shot peening on the surface, which may have led to more heterogeneity, showing an increase in effective capacitance. Additionally, the exponent (n) decreases from 0.901 for the unpeened sample to 0.757 for the peened sample, indicating the deviation from the ideal capacitive behavior to more heterogeneous conditions due to the shot peening. The charge transfer resistance (R_ct_) was recorded to be 9754 Ω·cm^2^ and 12,400 Ω·cm^2^ for the unpeened and peened samples, respectively. This rise in Rct indicates a lower rate of electrochemical reaction at the electrolyte/material interface, reflecting a more protective passive film and reduced tendency for localized corrosion. This improvement can be attributed to the partial closure porosity and localized densification of the surface/subsurface layer induced by shot peening, which limits electrolyte ingress and hinders transport across the metal/electrolyte boundary. The Warburg impedance (W) also changed dramatically because of shot peening. Warburg impedance increases from 7.46 μsnΩ^−1^ cm^−2^ in the unpeened samples to 264.9 μsnΩ^−1^ cm^−2^ after shot peening, likely due to increased surface complexity and hindered diffusion paths resulting from the severe surface modification after shot peening deformation. Micro roughness, dimple microstructure, and deformation after shot peening could have led to this complexity and interaction between the electrolyte and the peened surface. In the porous PM alloy, the fitted Rpo term is interpreted as a combined resistance associated with the near-surface film and pore/constriction pathways rather than a uniform oxide integrity. The R_po_ shows a reduction from 6830 Ω·cm^2^ in the unpeened samples to 1311 Ω·cm^2^ in the shot-peened sample, suggesting that shot peening disrupted the outer layer integrity, which is possibly a result of roughening and structural changes in the porous network. This complex interplay between improved surface sealing and altered passive layer morphology ultimately defines the corrosion performance of shot-peened Alumix 321 PM alloys.

**Figure 15:**
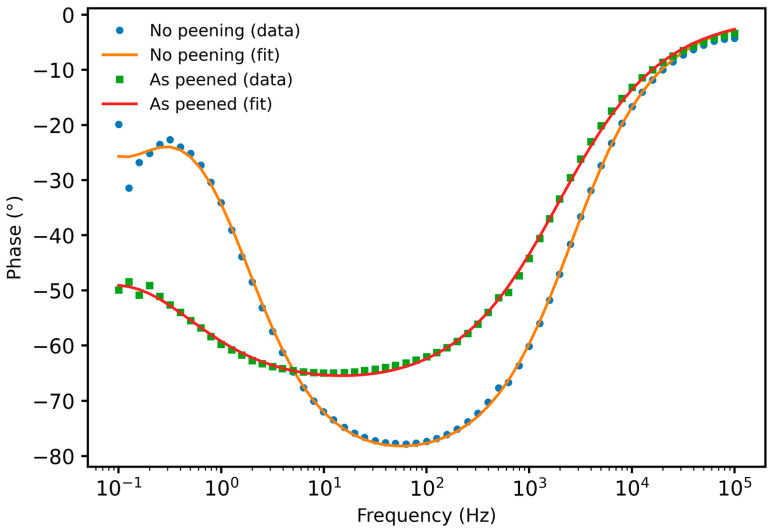
Bode plot of Alumix 321 PM alloy before and after shot peening.

The Bode phase plots shown in Figure 15 reveal a clear modification of the electrochemical response of Alumix 321 PM alloy after shot peening. The unpeened sample exhibits a more pronounced phase minimum (approaching 80°) at intermediate frequencies, indicating a dominant capacitive response associated with a relatively heterogeneous surface and active pore network. In contrast, the shot-peened condition shows a shallower phase minimum and a broader phase angle plateau, which is consistent with altered interfacial kinetics and increased surface heterogeneity resulting from near-surface deformation and partial pore closure. These changes suggest restricted electrolyte access to surface-connected porosity and modified charge transfer behavior after peening in agreement with the reduced corrosion current density and increased charge transfer resistance observed electrochemically. The equivalent circuit fits closely reproduce the experimental phase response across the full frequency range with good fit quality.

### 3.4. Characterization of the Corroded Samples

**Figure 16:**
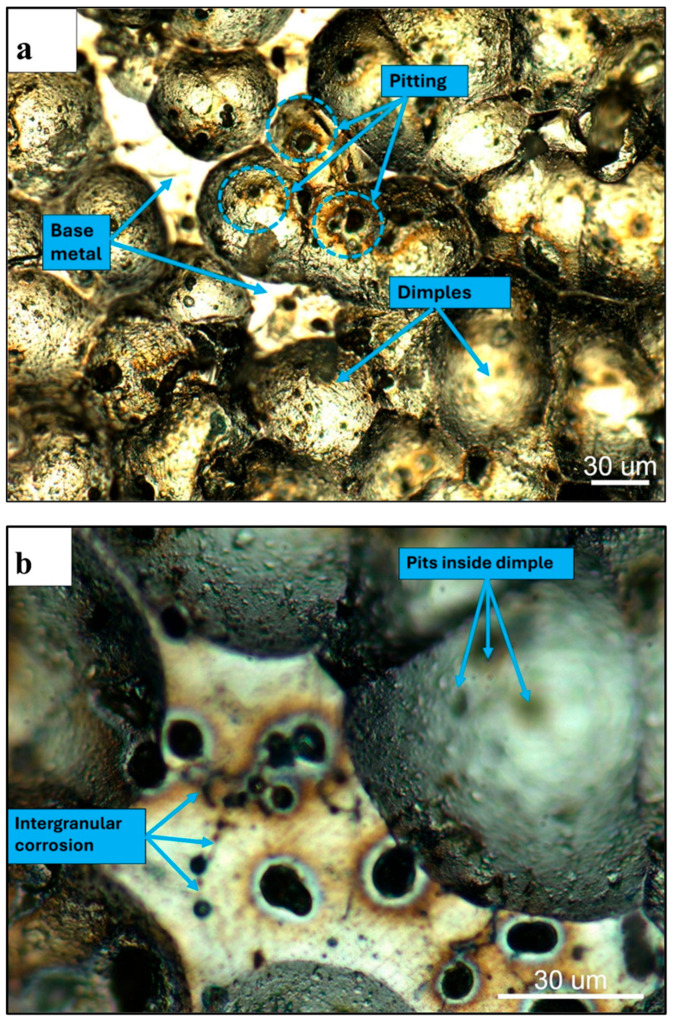
Optical micrographs of corroded surface of shot-peened Alumix 321 PM alloy in 3.5 w% NaCl solution: (**a**) low magnification shows dimple structure and undeformed islands; (**b**) high magnification shows intergranular corrosion attack. Exposure time is 24 h.

Figure 16a presents the optical micrograph of the corroded surface of the shot-peened Alumix 321 PM sample. Ideally, the shot peening process is expected to uniformly impact the entire surface, resulting in full coverage. However, the micrograph reveals the presence of isolated regions, “islands”, of undeformed material, indicating incomplete peening. These unpeened areas suggest that achieving 100% surface coverage was not feasible in this case. Image analysis confirmed a coverage of approximately 95%. These strain-free regions are likely to exhibit different electrochemical behavior compared to the plastically deformed zones, introducing heterogeneity that could influence the overall corrosion response of the alloy. Notably, visible pitting was observed within these undeformed regions, as well as within the dimpled features generated by peening. However, corrosion within the dimples appears comparatively minor, supporting the hypothesis that the peening induced compressive stresses and potential pore closure, which may enhance localized corrosion resistance. The high-magnification optical micrograph shown in Figure 16b provides clearer evidence of this phenomenon, showing pits both within the dimples and on the unaffected surface. This suggests that, while shot peening improves surface characteristics, incomplete coverage may still allow for preferential localized attack in unpeened regions.

**Figure 17:**
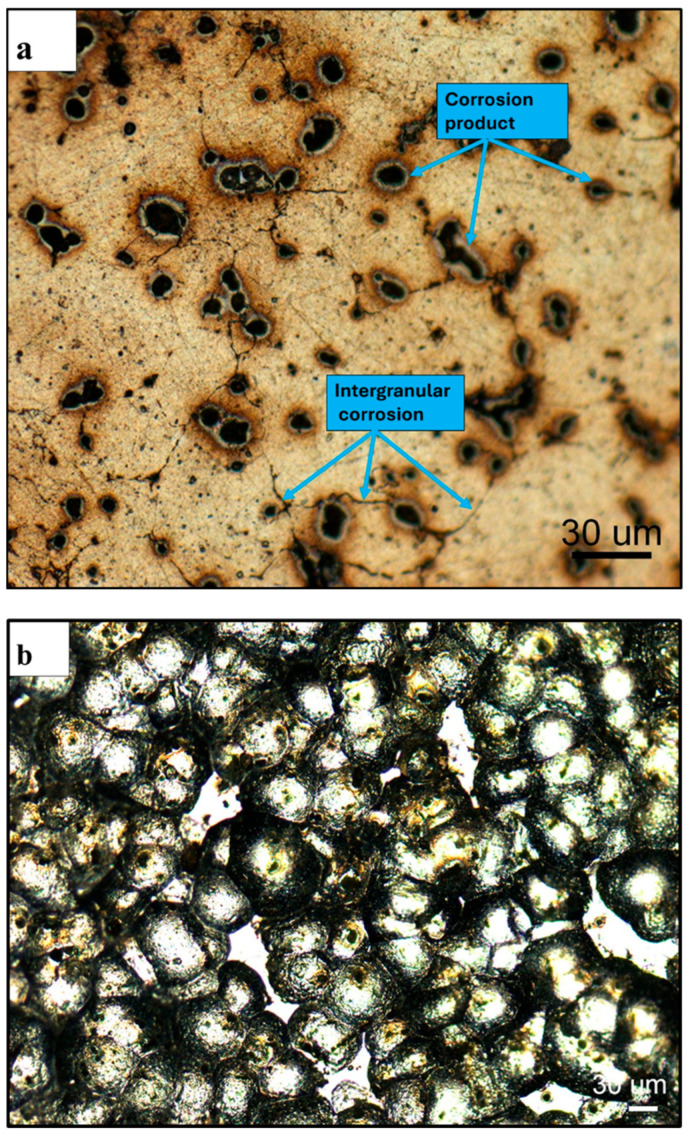
Optical micrograph of corroded surface of Alumix 321 PM alloy in 3.5 w% NaCl solution: (**a**) unpeened; (**b**) peened. Exposure time is 24 h.

Figure 17 shows the corroded surfaces of the as-sintered and shot-peened Alumix 321 PM samples. Shot peening appears to be effective in reducing the number of corrosion sites, a finding supported by the observed decrease in corrosion current density following the treatment. This improvement may be attributed, in part, to the partial collapse of interconnected pores during peening, which likely sealed some previously open pathways and reduced the overall surface area exposed to the electrolyte.

**Figure 18:**
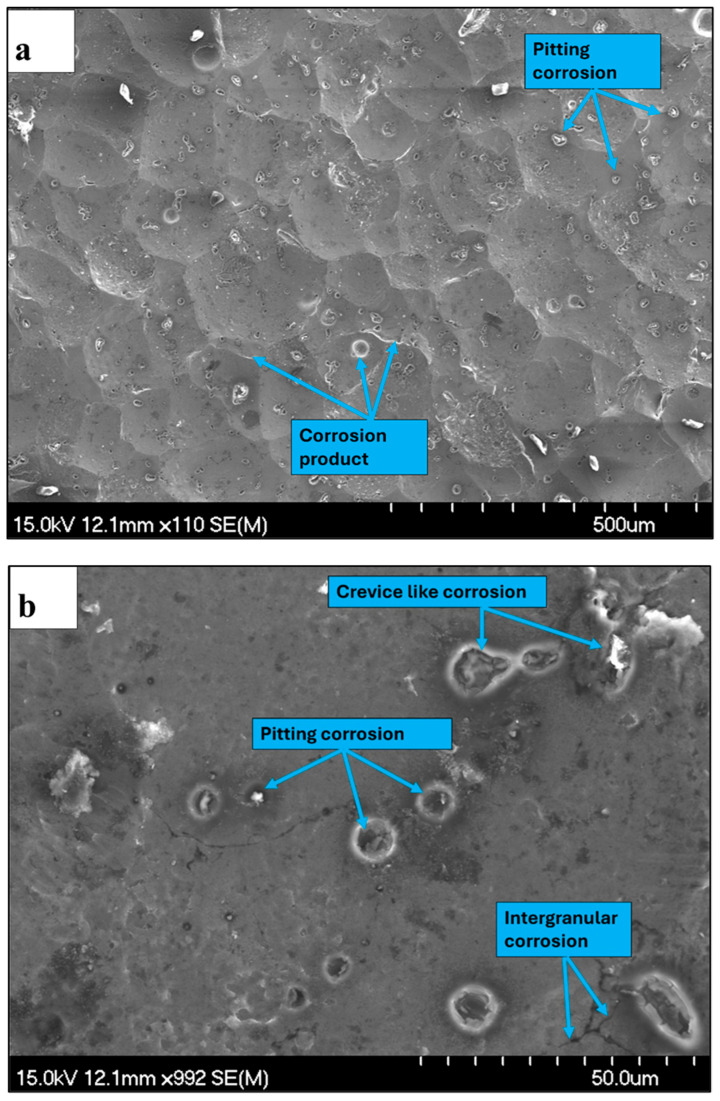
Corrosion morphology of shot-peened Alumix 321 PM alloy: (**a**) low magnification; (**b**) high magnification shows pitting, crevice and IGC. Exposure time is 24 h.

Figure 18a,b present low- and high-magnification SEM micrographs of the corroded Alumix 321 PM alloy after shot peening. The higher magnification image reveals rounded pits on the surface, some of which are located within the dimples created by peening. Additionally, evidence of corrosion within larger interconnected pores indicative of crevice-like attack is present, alongside signs of intergranular corrosion (IGC).

**Figure 19:**
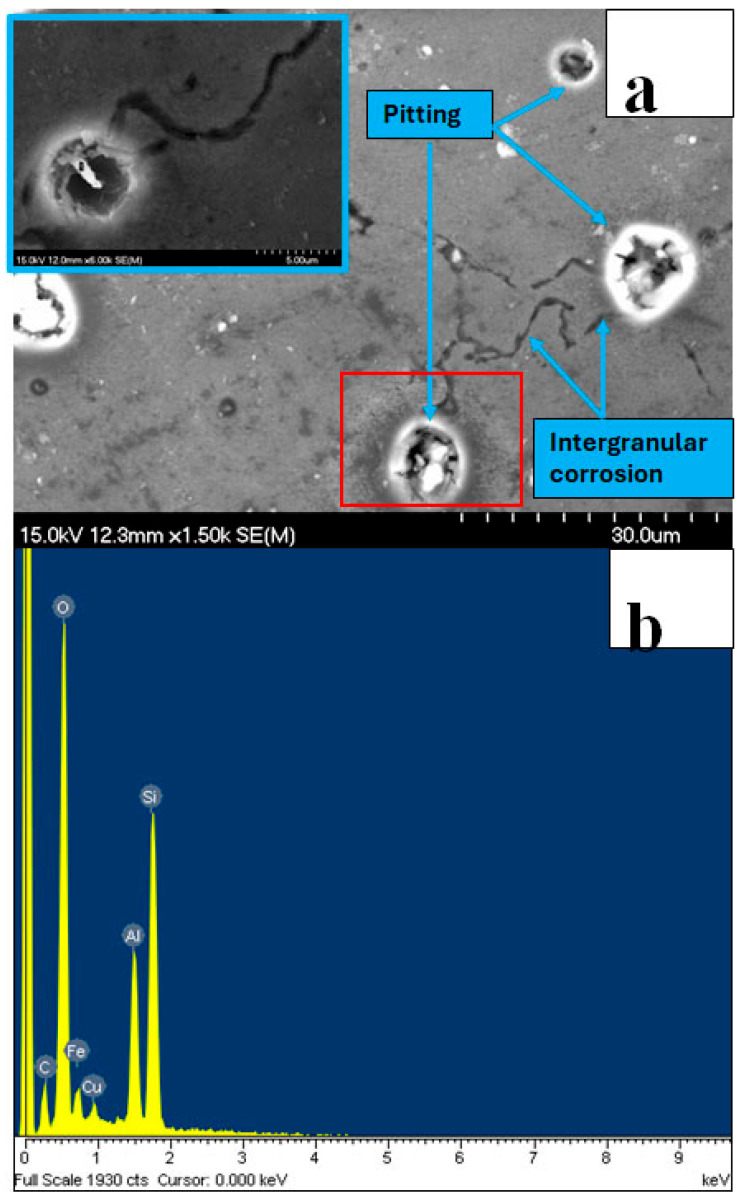
Corrosion morphology of Alumix 321 PM alloy: (**a**) pitting morphology; (**b**) EDS analysis of iron-containing particles. Exposure time is 24 h.

To gain deeper insight into the corrosion behavior of the Alumix 321 PM alloy, samples were immersed in a 3.5 wt.% NaCl solution and subsequently examined. Detailed surface analysis revealed fragmentation of the oxide layer. EDS confirmed that the detached fragments were enriched in iron. Further SEM examination performed on gently polished surfaces showed that pitting predominantly initiated in the vicinity of second-phase particles containing iron, silicon, and copper. These results indicate that pitting corrosion is characterized by the formation of hemispherical pits nucleating at or near Fe-rich intermetallic particles. The pit initiation is attributed to localized micro-galvanic corrosion cells between these cathodic particles and the surrounding α-aluminum matrix. Representative pitting morphologies are shown in Figure 19a, while Figure 19b presents the corresponding EDS elemental maps, confirming the association between pit formation and Fe-containing phases.

**Figure 20:**
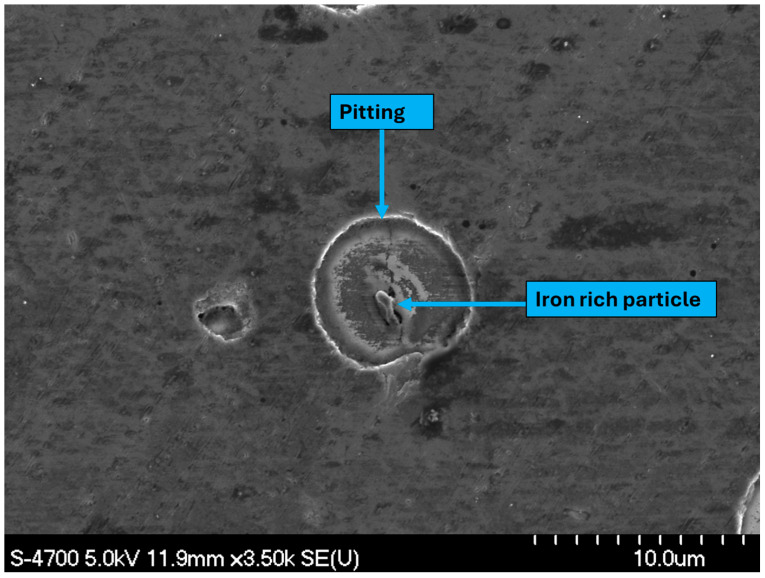
SEM micrograph shows corrosion pit formed around iron-rich particles in wrought AA 6061 alloy. Exposure time is 24 h.

**Figure 21:**
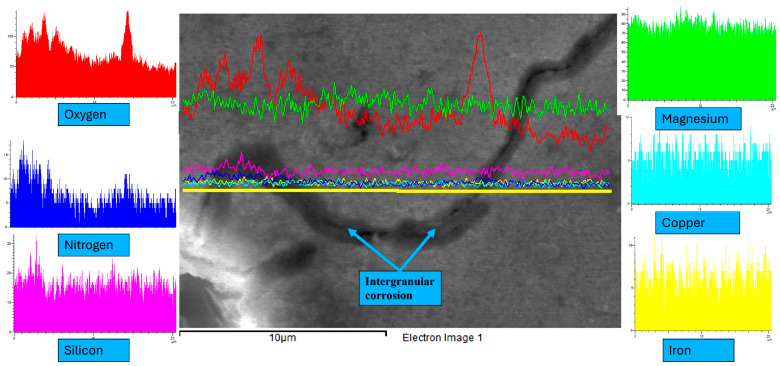
Line scan mapping shows corrosion at the grain boundary of Alumix 321 PM alloy. Exposure time is 24 h.

A similar analytical approach was employed to investigate the pitting behavior of the wrought AA 6061 alloy. As shown in Figure 20, pits were likewise observed to form preferentially around iron-rich second-phase particles. Despite differences in processing route and microstructural characteristics, both Alumix 321 PM and AA 6061 alloys exhibited pitting corrosion governed by the same micro-galvanic corrosion mechanism involving Fe-rich intermetallics and the aluminum matrix. Notably, no signs of IGC were observed in the AA 6061 samples. In contrast, the Alumix 321 PM samples exhibited clear features of intergranular corrosion. Figure 21 illustrates SEM evidence and elemental line mapping highlighting the IGC attack. The presence of IGC alongside pitting indicates that the PM alloy is susceptible to multiple corrosion modes in contrast to AA 6061, which only showed pitting corrosion.

**Figure 22:**
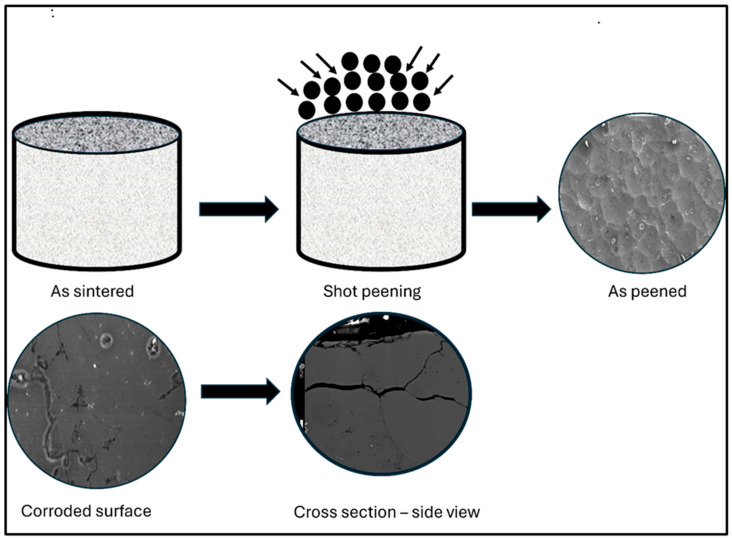
Schematic diagram shows the steps for examination of a cross-sectional sample.

**Figure 23:**
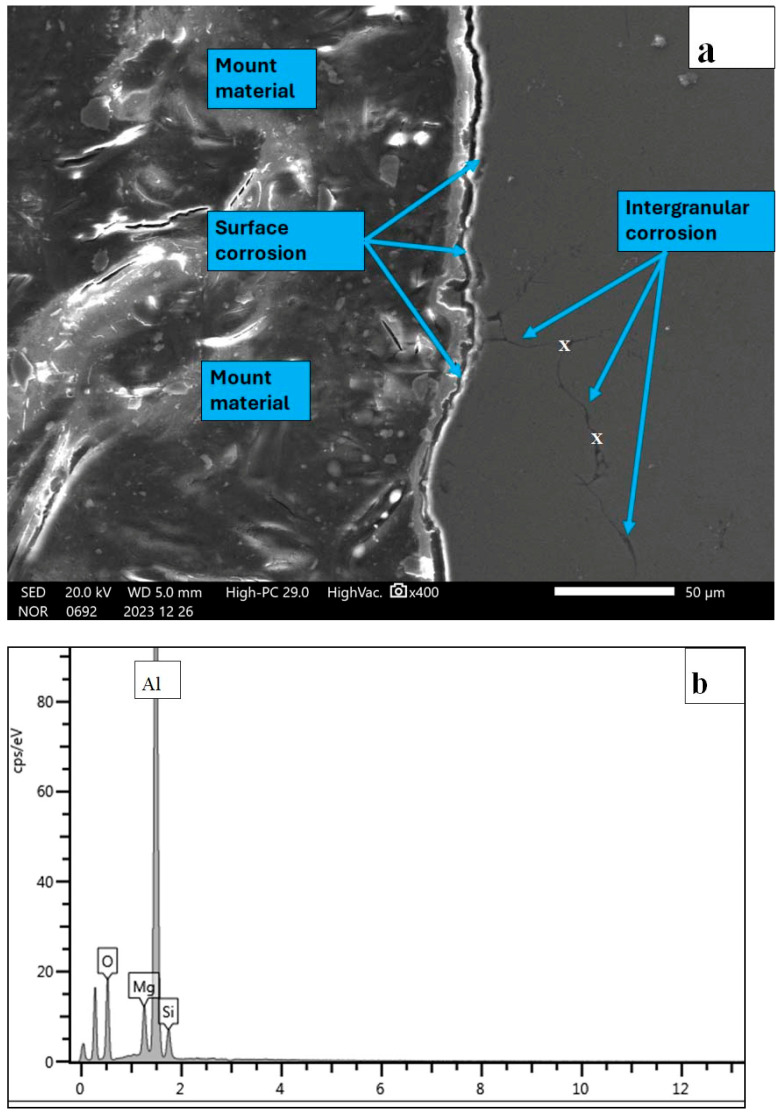
(**a**) SEM micrograph through a cross-section of corroded Alumix 321 PM alloy; (**b**) EDS spectrum acquired from the inter granular zone denoted by X.

For further clarification of the corrosion mechanism, selected corroded Alumix 321 PM samples were sectioned, mounted in epoxy, and carefully polished to reveal the cross-sectional morphology. Figure 22 presents the schematic of the cross-sectioned sample, while Figure 23a shows the microstructural features of the cross-section after corrosion.

The micrograph illustrated in Figure 23a shows a cross-sectional view of the shot-peened Alumix 321 PM alloy after exposure to a 3.5 wt.% NaCl solution. A continuous corrosion layer was observed along the surface, extending to a depth of approximately 10 µm. The presence of this relatively thin corrosion layer indicates that shot peening effectively enhanced the surface integrity of the alloy by closing or sealing most of the open pores. As a result, the penetration of the corrosive medium into the subsurface region was significantly reduced, and the majority of the corrosion damage remained limited to the outer surface. Nevertheless, localized regions exhibited evidence of intergranular corrosion, extending to depths of approximately 50 µm along certain grain boundaries. This observation suggests that a small number of unsealed or partially closed pores may have persisted after the shot peening treatment, providing preferential initiation sites or transport channels that facilitated the ingress of chloride ions and subsequent attack along the grain boundaries. These defects likely acted as pathways for localized electrolyte penetration, promoting the observed intergranular corrosion despite the overall improvement in surface densification. EDS analyses was conducted at several points along the affected grain boundaries, as shown in Figure 23b. The spectra revealed elevated concentrations of oxygen and aluminum consistent with Al-rich oxide/hydroxide-type corrosion products along the corroded grain boundaries. This compositional evidence confirms that corrosion propagation occurred preferentially at these localized regions, likely driven by microstructural heterogeneities and residual surface pores that remained even after shot peening.

## 4. Conclusions

Shot peening at an intensity of 0.4 mm N produced a uniform dimpled surface microstructure on the Alumix 321 PM alloy, indicating localized plastic deformation and pore closure. This treatment increased surface roughness while significantly reducing the corrosion rate from 0.079 to 0.004 mmpy, highlighting a marked improvement in corrosion resistance. Electrochemical testing revealed a decrease in corrosion current density and a shift of both corrosion and open-circuit potentials toward more negative values. The stair step polarization technique, using a slower scan rate, confirmed superior pitting resistance in shot-peened samples compared to unpeened ones, offering a more representative assessment of corrosion behavior. Compared to the wrought AA 6061 alloy, the Alumix 321 PM alloy exhibited distinct corrosion mechanisms due to its porous powder metallurgy microstructure. Overall, shot peening proved highly effective in improving the corrosion performance of the sintered Alumix 321 PM alloy.

## Figures and Tables

**Table 1:** Chemical composition of Alumix 321 PM alloy and the counterpart alloy AA 6061.

Alloy Type	Element	Mg	Si	Cu	Fe	Bi	Sn	V	Cr	Al
Alumix 321	Wt-%	1.31	0.5	0.32	0.10	0.01	0.03	0.01	----	Bal
AA 6061	Wt-%	0.93	0.61	0.30	0.34	0.0185	0.01	0.0084	0.07	Bal

**Table 2:** Corrosion parameters of the investigated alloys.

Corrosion Parameter	OCP (V vs. SCE)	E_corr_ (V vs. SCE)	E_pit_ (V vs. SCE)	E_pit_-SSP (V vs. SCE)	Corrosion Current	Corrosion Rate (mmpy)
Alloy					Density (A·cm^−2^)	
AA 6061	−0.736 ± 0.004	−0.714 ± 0.003	−0.714 ± 0.003	-------	1.77 × 10^−6^	0.019
Alumix 321 (unpeened)	−0.753 ± 0.005	−0.719 ± 0.016	−0.714 ± 0.004	−0.711 ± 0.05	7.86 × 10^−6^	0.079
Alumix 321 (shot-peened)	−0.769 ± 0.020	−0.762 ± 0.025	−0.716 ± 0.011	−0.690 ± 0.03	3.20 × 10^−7^	0.004

**Table 3:** Fitting parameters for Alumix 321 PM and shot-peened Alumix 321 PM alloy.

Material	R_s_ Ω cm^2^	R_po_ Ω cm^2^	CPE		R_ct_ Ω cm^2^	CPE		
			Ypo S·s^n^·cm^−2^	n		Yct	n	W S·s^n^·cm^−2^
Unpeened Alumix 321 PM	6.92	6830	24.42	0.901	9754	281.3	0.991	7.46
Shot-Peened Alumix 321 PM	7.98	1311	155.8	0.757	12,400	101.1	0.746	264.9

## Data Availability

The original contributions presented in this study are included in the article. Further inquiries can be directed to the corresponding author.
